# President’s Address*Read before the Chambers County Medical Society, January 17th, 1899.

**Published:** 1899-03

**Authors:** Benj. F. Rae

**Affiliations:** Lafayette, Ala.


ATLANTA
### Medical and Surgical Journal.

N.S. Vol. XVI. MARCH, 1899. No. 1.

DUNBAR ROY, A.B., M.D., EDITOR. M. B. HUTCHINS, M.D., BUSINESS MANAGER.

### ORIGINAL COMMUNICATIONS.

### PRESIDENT'S ADDRESS.*

By BENJ. F. RAE, Sr., M.D.,
Lafayette, Ala.

I have seen it stated somewhere, that whatever *must* be done, *can* be done. Whether this is true or not in every instance, it is certain that, whenever it becomes necessary to do anything worth doing, it is our *duty* to make the effort, although we may not in every instance feel fitted to accomplish the task.

There is such an endless variety of great and grand topics presented to us by our noble profession, that we should find no difficulty in selecting a subject suited to our capacity, and to which we have given sufficient amount of care and attention to enable us to arrange, bring together, and mould into proper form all of its best points, so as to render them presentable for discussion. Fortunately, in this instance, the subject has been selected for my consideration, and saves me the necessity of having to select and decide for myself.

*Read before the Chambers County Medical Society, January 17th, 1899.

WI
AT464

Although there is but a single article suggested for our consideration, the discussion of it brings up and presents to the mind of the physician two branches of the healing art, viz.: that of *surgery* and that of *practical medicine;* and just here I will say, without fear of contradiction, that there is not a profession so progressive as that of medicine—using the term in its broadest sense. Surgery, one of its most important branches, is rapidly approaching that of an exact science, and although surgery and practical medicine are steadily becoming more and more distinctly separated, and though *not the same,* yet they *must always* go hand in hand, and *ever remain* as inseparable as were the famous Siamese Twins. Neither one can rise or fall, exist or *live,* without the other.

Surgery is the treatment of effects; medicine the treatment of causes. *Effects* are plain and open before our eyes, and are seen with the trained eye and treated with the dexterous hand of the surgeon; while *causes* must be deduced as a consequence through a process of reasoning, taught us by close attention and experience.

The progressive surgeon must, necessarily, give a good share of his attention to therapeutics, or to some part of this branch of practical medicine, so as to be up-to-date or acquainted with the best and most approved remedies and their application to such morbid conditions as the surgeon is more especially called to treat. This calls to our mind that important class of remedies known as antiseptics and disinfectants, and many things under these headings, which only the surgeon is expected to enumerate. The attentive study of this class of remedies and the science of bacteriology, is to the surgeon of the present day a *ne plus ultra.* No one calling himself a surgeon can afford to neglect *any one* of the many points involved in antiseptic surgery. He who is negligent in one of these important steps will find himself in a short time left out of sight.

We come now to the consideration of the article suggested by my friend, Dr. Hudson, my experience with which I should arrange and bring before our County Society, namely, creosote. I will here state that I have been using this good old remedy from the immediate commencement of my practice in 1842.

After reading medicine two years (studying it assiduously expresses it more truly), I went to Philadelphia and remained two years, a private pupil the entire time under the great and good Dr. Robley Dunglison, and as soon as I returned home from old "Jeff," commenced practice, and from the first day, indeed the *first case* of any that I treated was a foul, sloughing, fetid and neglected ulcer, or wound caused by the bite of a large cur-dog. When called to this case, I found that the muscles of the calf of the leg had sloughed deeply, the fetid ulcer not less than three by four inches in extent. It had been going on nearly three months when I was first called to it. Knowing that the word creosote meant flesh-preserver, from the two Greek words *kreas*, flesh, and *soter*, preserver, I was not long, then and there, on deciding upon my plan of treatment; and I will here state that the treatment was just such *as I would use now*, and such as I have been using ever since that day. I make this assertion, and, if it were necessary, there are many who will testify to the truth of what I say. The treatment was antiseptic then, and I have used antiseptics ever since that time, now fifty-six years. The treatment in the above case was to cleanse the parts with *hot* water and soap, and apply "creosote-water" of about the strength of three or four drachms, more or less, to the pint of water; this being varied as to its strength. This application was continued, and from the first moment of its use the intolerable fetor disappeared, not to return, and the sloughing was arrested. This was in the case of a very irritable old woman of about eighty. Of course attention was given to her general health; in fact, such as we use now. In the use of water in all such cases, I use it as hot as the patient can bear, and am governed by feelings of patient, usually regardless of the nature or condition of the ulcer, or wound, as the case may be. I increase the temperature as the patient can bear it, with the same ratio of improvement in the progress of cure.

As stated, I used creosote at the commencement of my practice, and up to this day, and value it as highly now as I do any antiseptic in use.

There is not a physician, perhaps, during all this time, who has been with me in consultation, or simply in my company for only a short time, who has not observed that creosote was a favorite remedy with me, if there were any references to the use of it. The books *now* give all the information needed upon it, but fifty years ago there was but little information to be obtained concerning its properties.

During my pupilage two years under that good man Dr. T. W. Grimes, in the old town of Greensboro, Ga., the town of our nativity, we never prescribed it, nor did I ever see it used during the two years that I constantly attended the great Blockley Hospital, in Philadelphia, and I repeat that I had never seen or heard of its being used as an antiseptic application previous to my use of it, as stated.

When I was asked by my friend, Dr. Hudson, to write up "this article," I could not bring to mind but *one living person* whom I supposed to be acquainted with the facts of the case stated above, and wrote to this old woman, now eighty-five years old, to ascertain if she remembered this certain case and the remedy I used. She promptly replied, under date of March 30th, 1895: "I remember everything connected with the case which you mentioned in your letter, and that you used creosote-water, and I remember that I myself gave it the name of *smoky-water*, and it was in the spring of 1842 that you treated that bad leg." I have now her letter.

I will state that the first case of tubercular phthisis in which I gave creosote internally was in 1849, and I had not, up to this time, read or heard of its being used by any one for this disease. I remember all the circumstances connected with this case as distinctly as if they had occurred to-day. I have often given it in phthisis since my first experience with it, but have not time now to say more concerning it, but will at some other time. I will merely mention that the books and journals which I now see recommending it give smaller doses than I have been giving.

There are other diseases in which I use creosote, and can accomplish more with it than with any other remedy with which I have any knowledge or experience. There are diseases caused by microbes, which, before I had seen or heard the word bacteria, I called in my young days "fungous diseases," for I believed then that there were diseases produced in a variety of forms by microscopic seeds of fungi, as I then called them ; these seeds germinating wherever they fall upon a soil suited to the promotion of their growth and maturity ; some exterior, others interior, of the system, entering by inhalation and by absorption from food and drink introduced by the digestive passages.

In this connection, I will read you an extract from an article of mine which is published in the *Southern Medical and Surgical Journal* for March, 1852 :

. . . "The term aphthæ, which is sometimes applied to thrush, is incorrect, for the very word itself leads to the idea that there is inflammation, which is true of aphthous affections, but not of thrush. Both may exist simultaneously, as I have myself seen, but they are far from being one and the same disease—one being a vesicular affection, the other a *genuine parasitic deposit* (italics in the original), and this constitutes the true pathology (if you will allow the expression) of thrush . . . ."

. . . "The opinions expressed by Dr. Rea in the foregoing essay, were at variance with the views of some of the members of the society, but, on account of the intense cold, it was received without discussion . . . ."

When I wrote the article from which I have just read, I had never seen or read a book calling thrush a parasite. I had studied this matter for some years previous to writing it, and had mentioned it to others, calling it a fungus or spore or of this nature. I had also removed some of the deposit of thrush and had grown it on other substances.

The members of the society were "at variance" with my opinion, because they could not accept my views on the disease being due to a parasite.

In this same article I recommend as the best treatment for this parasitic disease, six, eight, or even more grains of nitrate of silver to the ounce of water, to be applied to these deposits.

I had thought much on this subject then, and talked to many intelligent men, both in the profession and with many who were not, but could get no information such as I needed, and such as you may believe was very scant fifty-six years ago, upon this subject at any rate, within my reach.

The use of creosote as an antiseptic application was as truly original with me as was the theorem of the renowned Archimedes that when a body is immersed in water it looses a portion of its weight exactly equal to the weight of the water displaced, and by this discovery he deduced the rule for finding the specific gravity of solids. I will remark that the same may be said of the use of creosote in phthisis. If it had ever been given or used by any one before I gave it, I did not know it.

I will remind the younger men of the profession that it is well enough to bear in mind that any "particular medicine" in their hands may not inaptly be compared to a tool in the hands of a mechanic; one mechanic may, with a certain tool, do a first-class job, to use the vulgar phrase, when another, with the identical tool, would make a botch, a mere clumsily patched job. Now, when you get what you find to be a good remedy in your hands, note carefully its effects in every case, and under every form and condition watch its effects when given by others, if convenient to do so, and you will find that, by long habit and strict observation, you will acquire a control over some diseases that would not yield to the same remedies carelessly or indiscriminately used by others. Those of us who have had more experience, and from a longer habit of observing closely the effects of our remedies, have, in many instances, seen our best remedies *sadly abused*, and as often, perhaps, *the patient*, if we dared to even think so. Young physicians do not, as a rule, devote sufficient time to therapeutics—that branch of practical medicine which should be the delight of all who pursue the science of medicine as a profession, and not as a trade, and my observation is that, more especially during the last decade, the neglect in this line of study has been lamentable. This is the especial branch of our profession that gives us the tools to work with, and should be well studied. We should bear in mind that nature is not always competent to meet every emergency, or we would need to have no doctor, so we should be prepared to approach disease with both eyes open and not like the blind man with a club, who hears disease and nature fighting, and steps up to separate them and boldly makes his blow; if he strikes nature, he kills nature; if he strikes disease, he kills disease.

It seems to be the case now that some of our good old remedies are giving up their virtues, or at any rate are being put aside to make room for the many new ones which are coming in so rapidly and in such numbers that the busy doctor would have much to do if he could only keep count of their *number* and *names*, not to say anything of the wonderful curative properties belonging to them. I think that I am as true to take good wherever I find it as the most of those who try to be progressive in our profession, and to be kind and respectful to all "new-comers," wishing them success, and, as far as I am able, observing their effects with a keen interest, but I most respectfully say that I have long since become assured that the vast majority of them are passing or have passed already away, not again to be heard of, and thus we may expect it to continue. Few out of the immense number *come to stay.* This alone, however, should not discourage us, for if we get even *one* out of twenty that is *better* than any other of its class that we have, it is surely worth all the time and labor given by our faithful and hard-working investigators. Many of our best remedies are from—but I am about to get off the subject, and must resume it at some other time—for I have extended this article to twice the length I intended when I commenced it.

